# Gasdermin-D and Caspase-7 are the key Caspase-1/8 substrates downstream of the NAIP5/NLRC4 inflammasome required for restriction of *Legionella pneumophila*

**DOI:** 10.1371/journal.ppat.1007886

**Published:** 2019-06-28

**Authors:** Augusto V. Gonçalves, Shally R. Margolis, Gustavo F. S. Quirino, Danielle P. A. Mascarenhas, Isabella Rauch, Randilea D. Nichols, Eduard Ansaldo, Mary F. Fontana, Russell E. Vance, Dario S. Zamboni

**Affiliations:** 1 Department of Cell Biology, Ribeirão Preto Medical School, University of São Paulo, Ribeirão Preto, São Paulo, Brazil; 2 Division of Immunology and Pathogenesis, Department of Molecular and Cell Biology, and Cancer Research Laboratory, University of California, Berkeley, California, United States of America; 3 Howard Hughes Medical Institute, University of California, Berkeley, California, United States of America; Gifu University, JAPAN

## Abstract

Inflammasomes are cytosolic multi-protein complexes that detect infection or cellular damage and activate the Caspase-1 (CASP1) protease. The NAIP5/NLRC4 inflammasome detects bacterial flagellin and is essential for resistance to the flagellated intracellular bacterium *Legionella pneumophila*. The effectors required downstream of NAIP5/NLRC4 to restrict bacterial replication remain unclear. Upon NAIP5/NLRC4 activation, CASP1 cleaves and activates the pore-forming protein Gasdermin-D (GSDMD) and the effector caspase-7 (CASP7). However, *Casp1*^–/–^ (and *Casp1/11*^–/–^) mice are only partially susceptible to *L*. *pneumophila* and do not phenocopy *Nlrc4*^–/–^mice, because NAIP5/NLRC4 also activates CASP8 for restriction of *L*. *pneumophila* infection. Here we show that CASP8 promotes the activation of CASP7 and that *Casp7/1/11*^*–/–*^ and *Casp8/1/11*^*–/–*^ mice recapitulate the full susceptibility of *Nlrc4*^–/–^ mice. *Gsdmd*^–/–^ mice exhibit only mild susceptibility to *L*. *pneumophila*, but *Gsdmd*^–/–^*Casp7*^–/–^ mice are as susceptible as the *Nlrc4*^–/–^ mice. These results demonstrate that GSDMD and CASP7 are the key substrates downstream of NAIP5/NLRC4/CASP1/8 required for resistance to *L*. *pneumophila*.

## Author summary

Inflammasomes are multi-protein complexes that detect infection and other stimuli and activate the Caspase-1 (CASP1) protease. The effectors required downstream of NAIP5/NLRC4 to restrict bacterial replication remain unclear. Active CASP1 cleaves and activates the pore-forming protein gasdermin D (GSDMD) to induce inflammation and cell death. We have previously shown that CASP8 is activated by the NAIP5/NLRC4 inflammasome independently of CASP1 and functions to restrict replication of the intracellular bacterium *Legionella pneumophila*. Here, we show that CASP7 is activated downstream of CASP8 and is required for CASP8-dependent restriction of *L*. *pneumophila* replication in macrophages and *in vivo*. In addition, CASP7 is also activated by CASP1. Taken together, these results imply that CASP7 and GSDMD are the two key caspase substrates downstream of NAIP5/NLRC4. In support of this hypothesis, we found that mice double deficient in CASP7 and GSDMD are more susceptible than the single knockouts and are as susceptible as the *Nlrc4* deficient mice for restriction of *L*. *pneumophila* replication *in vivo*. Collectively, our data indicate that GSDMD and CASP7 are activated by CASP1 and induce cell death and restriction of bacterial infection. Therefore, GSDMD and multiple caspases (CASP1, CASP7 and CASP8) operate downstream of the NAIP5/NLRC4 inflammasome for restriction of infection by pathogenic bacteria.

## Introduction

Inflammasomes are multi-protein complexes that assemble in the cytosol of infected or damaged cells and initiate host defense by functioning as a platform for the recruitment and activation of caspase proteases [1,2]. The NAIP/NLRC4 family of inflammasomes is especially well-characterized and has been shown to be activated upon detection of specific bacterial proteins, such as flagellin, via direct binding to various NAIP family members [3]. Ligand-activated NAIPs recruit and co-oligomerize with NLRC4, which in turn recruits and activates Caspase-1 directly, or indirectly via the adaptor protein ASC. Recently it was shown that ASC can also recruit and activate Caspase-8 downstream of NAIP/NLRC4 [4,5].

The NAIP5/NLRC4 inflammasome was originally discovered as an essential host component to restrict intracellular replication of several bacterial pathogens, including *Legionella pneumophila*, the causative agent of a severe pneumonia called Legionnaires’ Disease [6,7]. NAIP5 binds directly to *L*. *pneumophila* flagellin [8–10], resulting in NLRC4 and CASP1/8 activation. Flagellin-deficient *L*. *pneumophila* evade detection by NAIP5/NLRC4 [11,12] and NAIP5-deficient or NLRC4-deficient cells or mice also fail to detect or restrict the intracellular replication of flagellated *L*. *pneumophila* [13–15], but the underlying effectors downstream of NAIP5/NLRC4 required for resistance to *L*. *pneumophila* remain unclear. Caspase-1 cleaves dozens of host proteins [16–18], but two key substrates suggested to participate in host defense are Gasdermin-D (GSDMD) (reviewed in [19]) and the pro-inflammatory cytokines interleukin-1β (IL-1β) and IL-18. Cleaved Gasdermin-D oligomerizes and inserts into the plasma membrane to form large pores [20,21], leading to release of IL-1β/-18, as well as to a characteristic form of cell death called pyroptosis. *In vitro*, pyroptotic cell death, but not IL-1β/-18, is believed to restrict the intracellular replication of *L*. *pneumophila* in macrophages, presumably by elimination of the intracellular niche required for bacterial replication. However, CASP1-deficient macrophages are only partially susceptible to *L*. *pneumophila* [4,22] and the CASP8 substrates that contribute to inflammasome-mediated host defense remain unclear.

Caspase-11 (CASP11) and Caspase-7 (CASP7) are additional caspases previously implicated in resistance to *L*. *pneumophila*. CASP11 detects *L*. *pneumophila* lipopolysaccharide (LPS) and triggers GSDMD cleavage to activate pyroptosis independent of NAIP5/NLRC4 activation. Although CASP11 is activated by *L*. *pneumophila* [23–26], CASP11 does not appear to play a major role in restricting bacterial replication in bone marrow macrophages, as *Casp11*^–/–^macrophages fully restrict *L*. *pneumophila* replication, and *Nlrc4*^–/–^macrophages, which still harbor functional CASP11, are fully permissive [27]. The lack of a discernable role for CASP11 in restricting *L*. *pneumophila* is likely due to a requirement for ‘priming’ signals to induce CASP11 expression, as well as to redundancy with the NAIP5/NLRC4 inflammasome.

CASP7 has also been reported to be activated downstream of flagellin detection and CASP1 activation by the NAIP5/NLRC4 inflammasome [28]. NAIP5/NLRC4-dependent CASP7 activation was reported to require CASP1 and, consistent with previous work [17], CASP7 was suggested to be cleaved directly by CASP1. In fact, *Casp7*^–/–^macrophages were reported to phenocopy the susceptibility of *Casp1*^–/–^macrophages, and CASP7 was thus proposed to be required for CASP1-dependent resistance to *L*. *pneumophila* [28]. Although GSDMD was not known at the time of this work, in retrospect it is surprising *Casp7*^–/–^cells would recapitulate the susceptibility of *Casp1*^–/–^cells given the clear role for GSDMD as a direct CASP1 substrate that is sufficient to mediate pyroptosis. Moreover, given that NAIP5/NLRC4 activates CASP8 [4,5,29], and that CASP8 cleaves CASP7 [30], it is surprising that CASP1 would be required for CASP7 activation.

Here we sought to define the key effectors downstream of the NAIP5/NLRC4 inflammasome that are required to restrict bacterial replication. Consistent with prior studies, we find that both CASP1 and CASP8 are activated by the NAIP5/NLRC4 inflammasome. Importantly, we find that mice doubly deficient in both enzymes fully recapitulate the susceptibility of NAIP5/NLRC4-deficient mice. We further find that CASP7 activation downstream of NAIP5/NLRC4 is mediated by CASP8 in addition to CASP1. Thus, mice singly deficient in CASP1, CASP7 or GSDMD are not fully susceptible to *L*. *pneumophila*, whereas *Casp7/1/11*^*–/–*^and *Gsdmd/Casp7*^*–/–*^mice phenocopy the full susceptibility of *Nlrc4*^*–/–*^mice to *L*. *pneumophila*. Taken together our results identify CASP7 and GSDMD as the key mediators downstream of NAIP5/NLRC4 inflammasome activation.

## Results

### CASP8 is activated in the absence of GSDMD and CASP1/11 for restriction of *L*. *pneumophila* replication in macrophages

**Fig 1:**
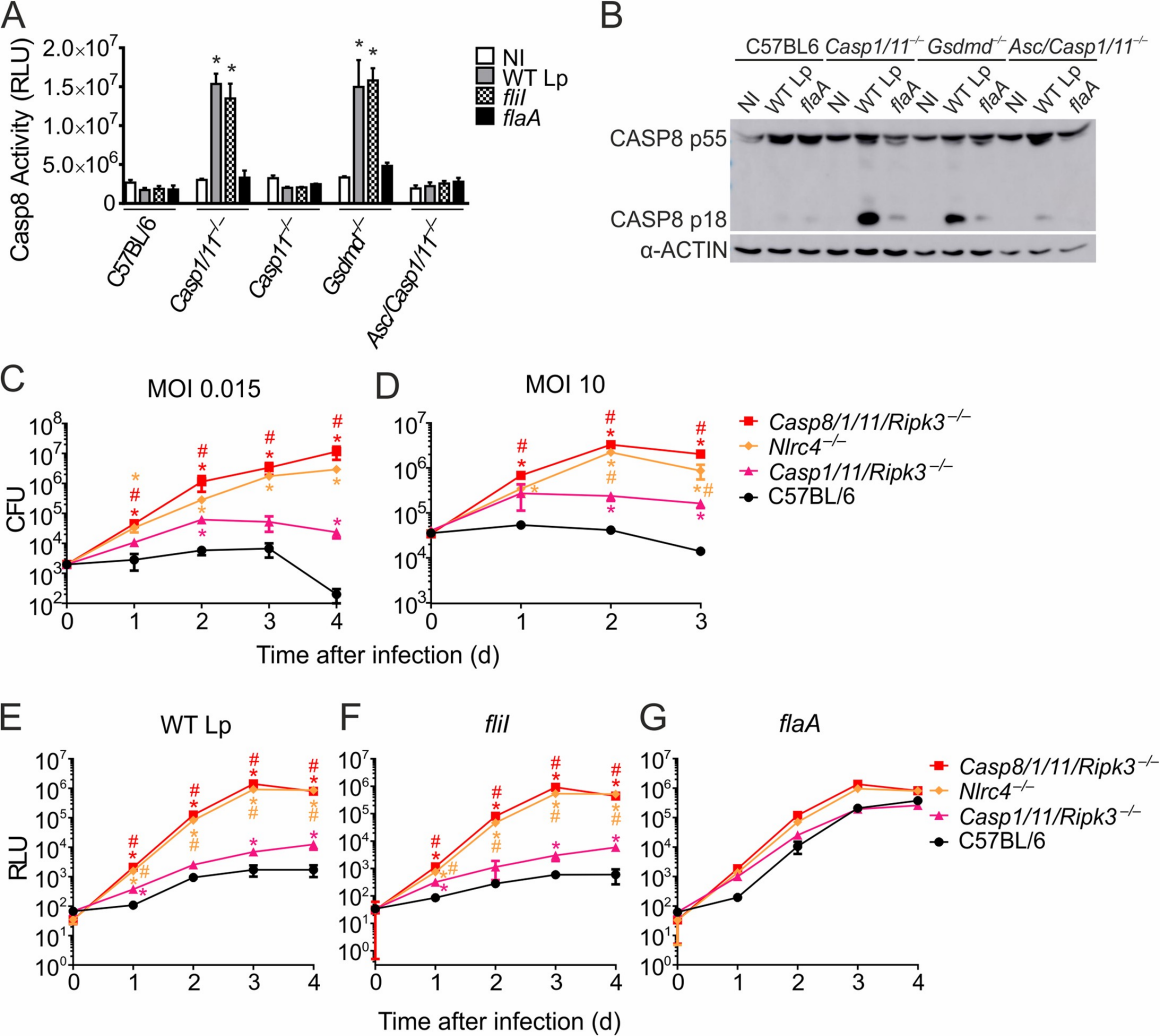
CASP8 is activated in the absence of GSDMD and CASP1/11 and it is important for restriction of *L*. *pneumophila* replication in macrophages. (**A**) Bone marrow-derived macrophages from C57BL/6, *Casp1/11*^*–/–*^, *Casp11*^*–/–*^, *Gsdmd*^*–/–*^and *Asc/Casp1/11*^*–/–*^mice were left uninfected or infected with wild type *L*. *pneumophila* (WT Lp; grey bars), *fliI* mutants (hatched bars) or *flaA* mutants (*flaA*; black bars) at an MOI of 10 for 8 hours and the activation of CASP8 was measured using the Caspase-Glo® 8 Assay kit. (**B**) Macrophages were infected at an MOI of 10 for 8 hours and the cleavage of CASP8 was measured by western blot using the anti-Casp8 and anti-α-actin antibody. (**C-G**) Macrophages from C57BL/6, *Casp1/11/Ripk3*^*–/–*^, *Casp8/1/11/Ripk3*^*–/–*^or *Nlrc4*^*–/–*^mice were infected with *L*. *pneumophila* and the bacterial replication was measured for up to 4 days of infection. (**C, D**) Cells were infected with wild type *L*. *pneumophila* at an MOI of 0.015 (**C**) or 10 (**D**) and bacterial replication was estimated by CFU counting. (**E-G**) Cells were infected with wild type *L*. *pneumophila* (**E**), *fliI* mutants (**F**) or *flaA* mutants (**G**) expressing luciferase at an MOI of 0.015 and bacterial replication was estimated by measuring the luminescence (RLU) of each well over 4 days of infection. *, *P*<0.05: compared to C57BL/6. ^#^, *P*<0.05: compared to *Casp1/11/Ripk3*^*–/–*^. Data are presented for one representative experiment of three (**A- D**) and four (**E-G**) experiments performed with similar results.

We have previously shown that CASP8 is activated in response to *L*. *pneumophila* infection when we silence GSDMD or in the absence of CASP1 [4]. To confirm these data, we infected macrophages deficient in GSDMD (*Gsdmd*^*–/–*^) with *L*. *pneumophila* and measured CASP8 activation using western blot and a substrate that detects CASP8 activity. We found that infection with wild type bacteria and *fliI* mutants (that express cytosolic flagellin, but do not assembly the flagellum), but not with *flaA* mutants, triggers robust CASP8 activation in *Casp1/11*^*–/–*^and *Gsdmd*^*–/–*^macrophages (**Fig 1A and 1B**). CASP8 did not appear to be robustly activated in C57BL/6 or *Casp11*^*–/–*^macrophages, possibly because these macrophages undergo rapid pyroptosis. In addition, CASP8 activation was not observed in *Asc/Casp1/11*^*–/–*^macrophages (**Fig 1A and 1B**). These data support previous findings indicating that CASP8 is activated in inflammasomes, particularly when CASP1 or GSDMD is absent, in a process that requires ASC [4,5,31].

Next, we tested the role of CASP8 in restricting *L*. *pneumophila* replication in macrophages in the absence of CASP1/11. We have previously shown that *Nlrc4*^*–/–*^macrophages are more permissive than *Casp1/11*^*–/–*^[22], implying that CASP1 is not the sole caspase activated by NAIP5/NLRC4. Thus, to discern a role for CASP8, we compared macrophages deficient in *Casp8/1/11/Ripk3* with those deficient in *Casp1/11/Ripk3*. Experiments were conducted in a *Ripk3* mutant background because *Casp8*-deficiency is embryonically lethal except in the absence of *Ripk3* [32]. We found that whereas *Casp1/11/Ripk3*^*–/–*^macrophages are slightly more permissive to *L*. *pneumophila* replication than the C57BL/6, the *Casp8/1/11/Ripk3*^*–/–*^cells are highly susceptible, similar to *Nlrc4*^*–/–*^macrophages. This was shown using both very low MOI (MOI = 0.015) and high MOI (MOI = 10) infections (**Fig 1C and 1D**). We also assessed bacterial replication in macrophages using a *L*. *pneumophila* strain stably expressing the *Photorhabdus luminescens luxCDABE (lux)* operon as described previously [33]. We generated a JR32 strain of *L*. *pneumophila* expressing the *lux* operon and detected robust bacterial replication in *Casp8/1/11/Ripk3*^*–/–*^and *Nlrc4*^*–/–*^macrophages (**Fig 1E**). In contrast, C57BL/6 and *Casp1/11/Ripk3*^*–/–*^macrophages were restrictive to bacterial replication (**Fig 1E**). This was observed using wild type JR32 *L*. *pneumophila* (**Fig 1E**) and *fliI* mutants (**Fig 1F**). As expected, isogenic *flaA* mutants expressing the *lux* operon robustly replicate in all macrophages used (**Fig 1G**). We also tested the importance of CASP8 for bacterial growth restriction using the wild type Lp02 strain of *L*. *pneumophila* and obtained comparable results (**S1A Fig**). As expected, flagellin mutants of Lp02 *L*. *pneumophila* effectively replicated in all macrophages used (**S1B Fig**), whereas *dotA* mutants were defective for intracellular replication (**S1C Fig**).

### In the absence of CASP1/11, CASP7 is activated downstream of CASP8 and accounts for macrophage cell death

**Fig 2:**
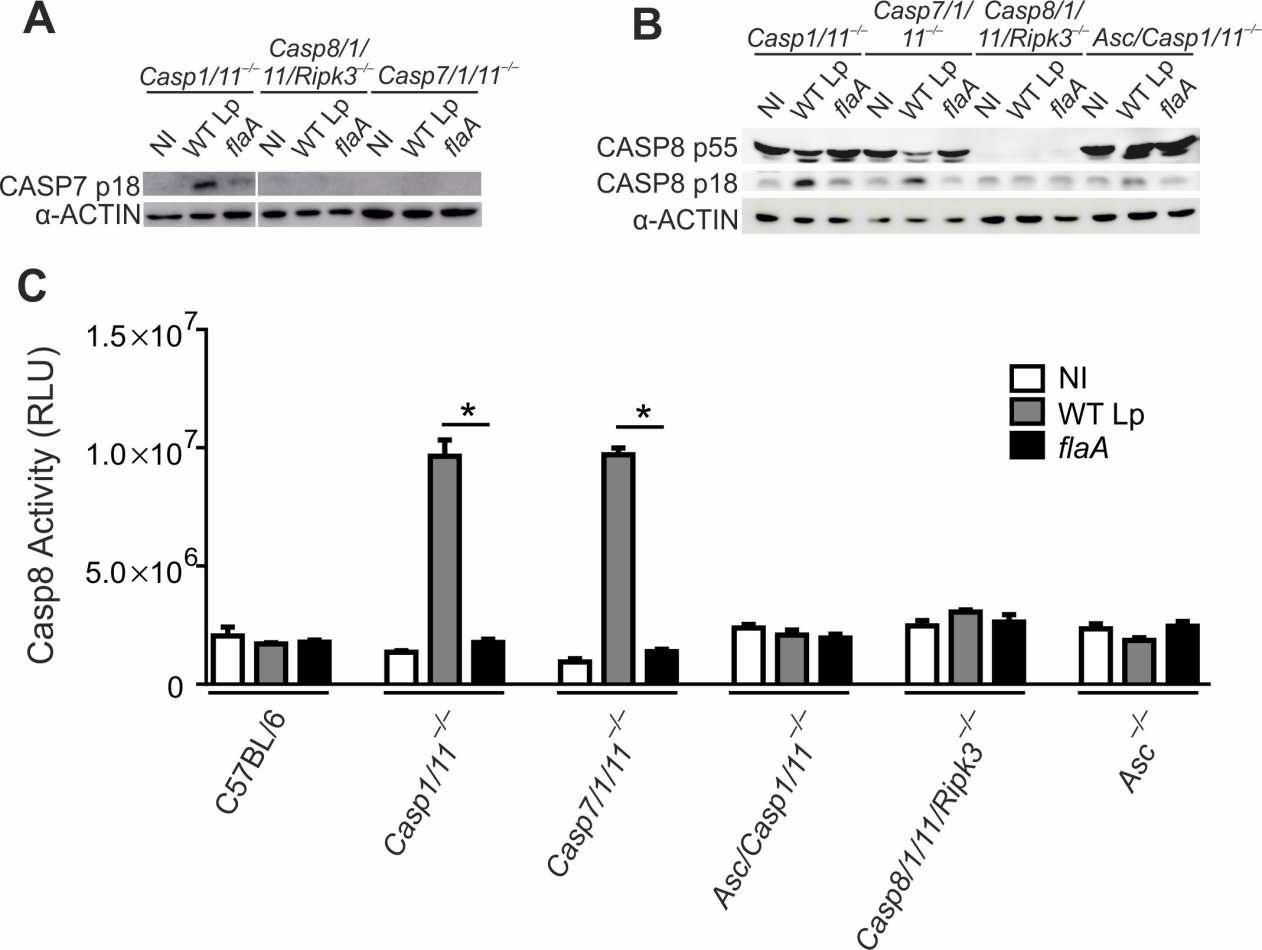
CASP7 is activated downstream of CASP8 in the NAIP5/NLRC4 inflammasome. Bone marrow-derived macrophages from *Casp1/11*^*–/–*^, *Asc/Casp1/11*^*–/–*^, *Casp8/1/11/Ripk3*^*–/–*^and *Casp7/1/11*^*–/–*^mice were left uninfected (NI, open bars) or infected with wild type *L*. *pneumophila* (WT Lp, grey bars) or *flaA* mutants (black bars) at an MOI of 10 for 8 hours. (**A**) Caspase-7 cleavage was measured by western blot using anti-Casp7 p18 and anti-α-actin antibody. (**B and C**) Caspase-8 cleavage was measured by western blot using anti-Casp8 p18 and anti-α-actin antibody (**B**) and Caspase-Glo 8 Assay kit (**C**). *, *P*<0.05. Data are presented for one representative experiment of four (**A**) and three (**B**) and two (**C**) experiments performed with similar results.

CASP7 was previously proposed to require CASP1 for activation downstream of NAIP5/NLRC4 [28]. However, since CASP7 is also known to be a substrate of CASP8 [30,34], we decided to re-assess CASP7 activation in CASP1-deficient cells where CASP8 is still active. We infected *Casp1/11*^*–/–*^macrophages with wild type *L*. *pneumophila* (or with *flaA* mutants as control) and assessed CASP7 processing by western blot using an antibody specific for CASP7 p18. We found that CASP7 is processed in response to flagellin in *Casp1/11*^*–/–*^macrophages (**Fig 2A**). In *Casp1/11*^–/–^cells, CASP7 processing in response to flagellin requires CASP8 because we detect no CASP7 p18 in Casp8*/1/11/Ripk3*^*–/–*^macrophages (**Fig 2A**). In this experiment, *Casp7/1/11*^*–/–*^macrophages were used to confirm the specificity of the anti-CASP7 p18 antibody (**Fig 2A**). Next, we used the same macrophages to test if CASP7 is required for CASP8 activation. By assessing CASP8 activation by western blot and by the chemiluminescent substrate, we found that CASP8 is activated in *Casp1/11*^*–/–*^and in *Casp7/1/11*^*–/–*^macrophages, but not in *Asc/Casp1/11*^*–/–*^or Casp8*/1/11/Ripk3*^*–/–*^macrophages (**Fig 2B and 2C**). These data indicate that, as expected, CASP7 is not required for CASP8 activation in the absence of CASP1/11. Experiments performed with immortalized macrophages confirmed that ASC but not CASP7 is important for CASP8 activation in the absence of CASP1/11 (**S2 Fig**).

**Fig 3:**
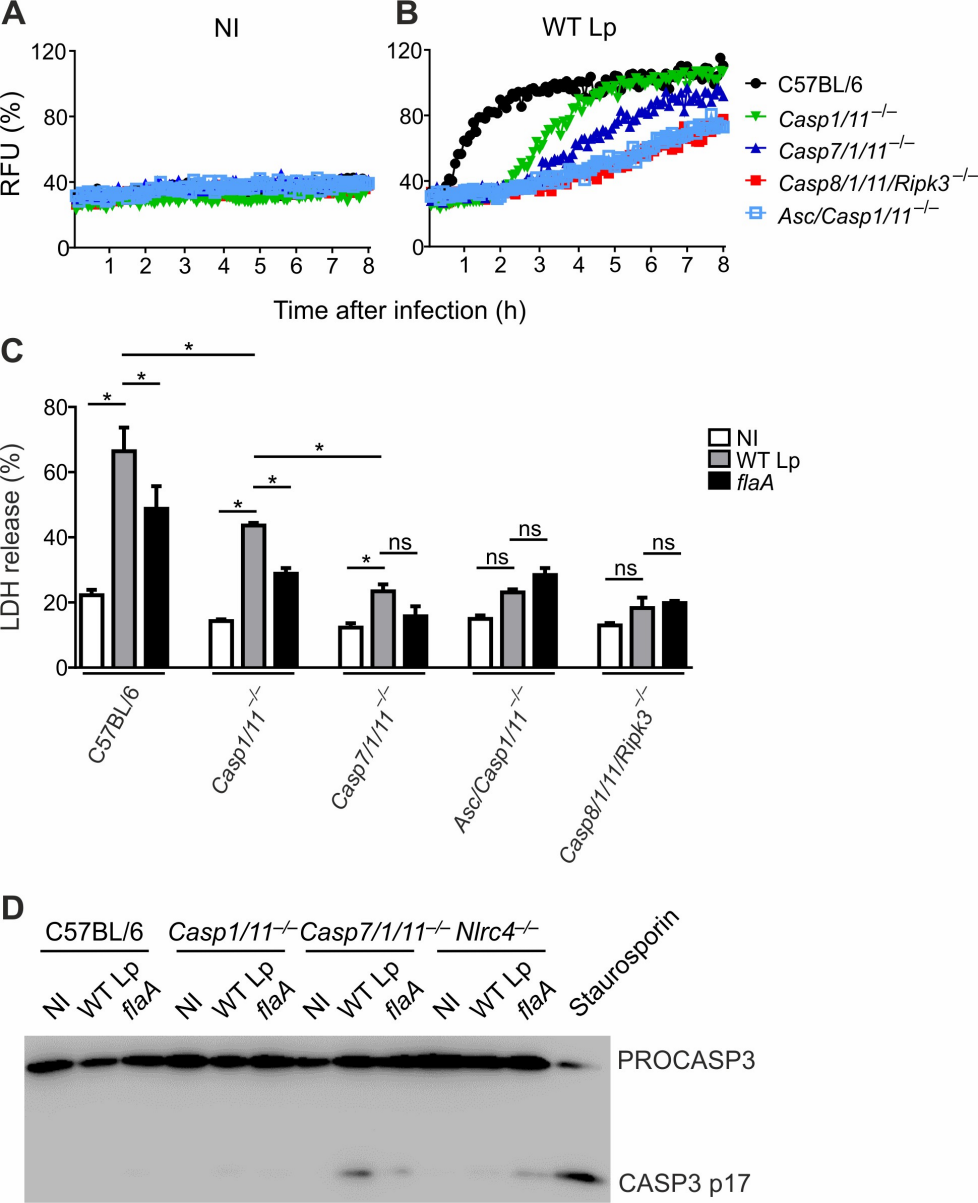
CASP7 accounts for pore formation and pyroptosis. Bone marrow-derived macrophages from C57BL/6, *Casp1/11*^*–/–*^, *Casp7/1/11*^*–/–*^, *Casp8/1/11/Ripk3*^*–/–*^and *Asc/Casp1/11*^*–/–*^mice were left uninfected (NI) or infected with wild type *L*. *pneumophila* (WT Lp) or *flaA* mutants at an MOI of 10. (**A and B**) Pore formation in uninfected (**A**) and WT Lp-infected (**B**) macrophages was assessed by Propidium Iodide (PI) uptake. Data are expressed as a percentage of total PI uptake (estimated using Triton-X100). (**C**) LDH release was assessed 7 hours after infection using CytoTox 96 Non-Radioactive Cytotoxicity Assay. (**D**) Lysates from C57BL/6, *Casp1/11*^*–/–*^, *Casp7/1/11*^*–/–*^and *Nlrc4*^*–/–*^macrophages were assessed for CASP3 cleavage using anti-Casp3 antibodies. Data are expressed as a percentage of LDH release induced by Triton-X100. Statistical significance was calculated using Student’s *t* test. *, *P*<0.05. ns: non-significant. Data are presented for one representative experiment of four (**A-B**) and two (**C-D**) experiments performed with similar results.

We and others have previously shown that *L*. *pneumophila* infection triggers pore formation and pyroptosis that is dependent on CASP1 and CASP11 [11,12,25,35]. We also showed that in the absence of CASP1 and CASP11, *L*. *pneumophila* induces pore formation dependent on flagellin, ASC and CASP8 [4]. Thus, we tested if CASP7 accounts for pore formation downstream of CASP8. We measured pore formation in real time by assessing the uptake of propidium iodide into the nuclei of permeabilized macrophages as described [25]. We confirmed that pore formation occurs in *Casp1/11*^*–/–*^macrophages in response to *L*. *pneumophila* infection (**Fig 3A and 3B**). The Caspase-1/11-independent pore formation is abolished in *Asc/Casp1/11*^*–/–*^and Casp8*/1/11/Ripk3*^*–/–*^macrophages and is reduced in *Casp7/1/11*^*–/–*^macrophages (**Fig 3B**). As previously demonstrated, Fig 3B shows that pore formation in wild type C57BL/6 cells is very robust because it occurs through CASP1 and CASP11 [25]. We also measured LDH release after infection as a readout for membrane leakage. As suggested by the pore formation assay, we found that LDH release occurs in C57BL/6 and *Casp1/11*^*–/–*^but not in *Asc/Casp1/11*^*–/–*^or Casp8*/1/11/Ripk3*^*–/–*^macrophages (**Fig 3C**). Although reduced, we still detected cell death in *Casp7/1/11*^*–/–*^cells in response to infection (**Fig 3C**). We measured CASP3 activation by western blot and confirmed that CASP3 is activated in response to flagellin in *Casp7/1/11*^*–/–*^cells, but not in C57BL/6, *Casp1/11*^*–/–*^and *Nlrc4*^*–/–*^macrophages (**Fig 3D**). These data suggest that CASP3 does not play a significant role downstream of NAIP5/NLRC4 inflammasome as previously reported [13–15], but can account to explain the modest levels of cell death detected in *Casp7/1/11*^*–/–*^cells. Collectively, these data suggest that in the absence of CASP1/11, macrophages are still able to respond to flagellin and trigger pore formation and pyroptosis via ASC and CASP8 and partially via CASP7.

### CASP7 activation downstream of CASP8 is important for restriction of *L*. *pneumophila* replication in macrophages and in vivo

**Fig 4:**
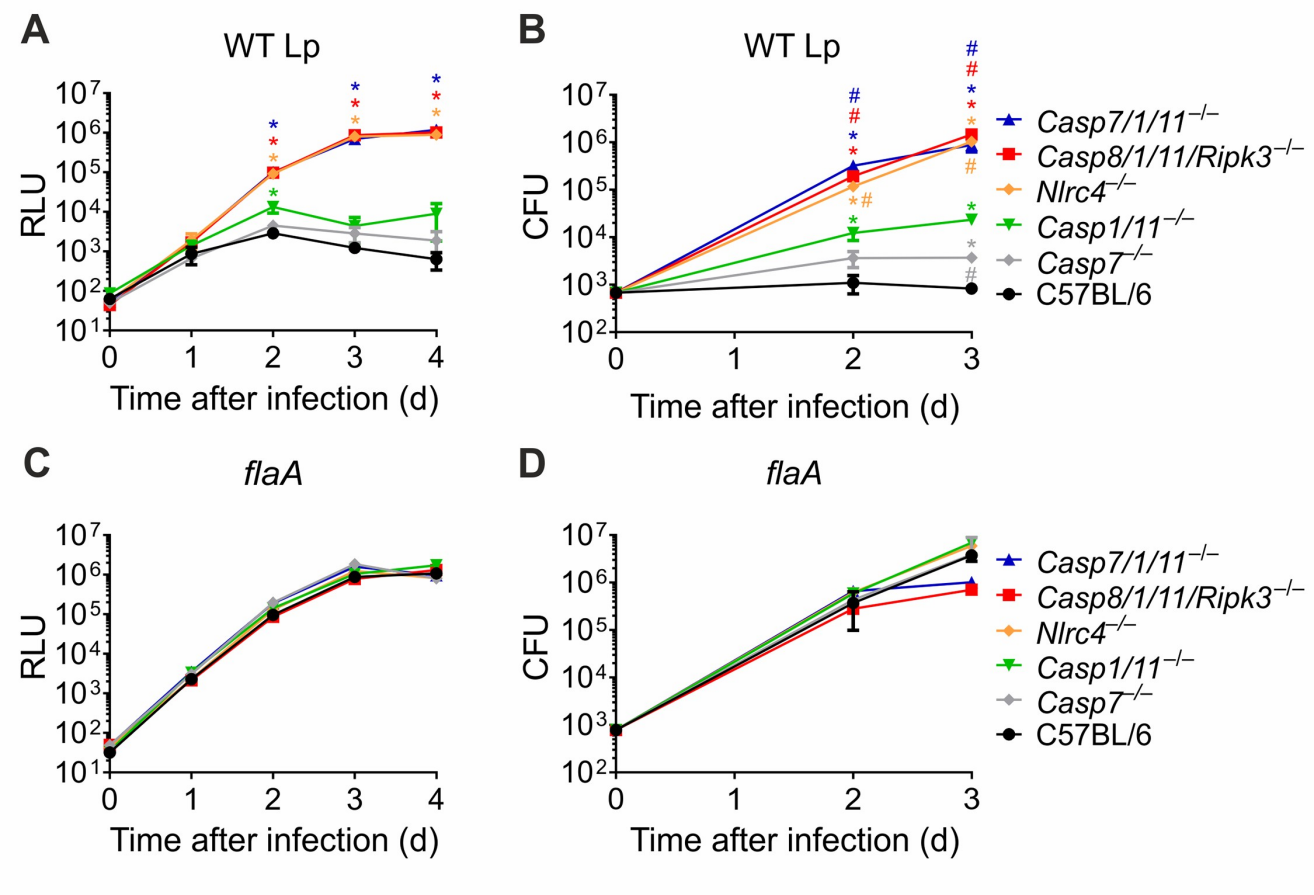
CASP7 is important for restriction of *L*. *pneumophila* replication in vivo in the absence of CASP1/11. Bone marrow-derived macrophages from C57BL/6, *Casp7*^*–/–*^, *Casp1/11*^*–/–*^, *Casp7/1/11*^*–/–*^,*Casp8/1/11/Ripk3*^*–/–*^and *Nlrc4*^*–/–*^mice were infected with wild type *L*. *pneumophila* (WT Lp, **A, B**) or *flaA* mutants (**C, D**). Macrophages were infected at an MOI of 0.015 and bacterial replication was assessed by measurement of luminescence (RLU) emitted by luciferase-expressing bacteria (**A, C**) or by estimating bacterial CFU (**B, D**). *, *P*<0.05: compared to C57BL/6. ^#^, *P*<0.05: compared to *Casp1/11*^*–/–*^. Data are presented for one representative experiment of four (**A, C**) and three (**B, D**) experiments performed with similar results.

Next, we tested the importance of CASP7 for restriction of *L*. *pneumophila* replication in macrophages. To address this we used macrophages from *Casp7*^*–/–*^single mutants and also from mice triple deficient for CASP7/1/11 (*Casp7/1/11*^*–/–*^). In contrast to a previous report [28], we found that *Casp7*^*–/–*^macrophages efficiently restrict the replication of wild type *L*. *pneumophila* as measured by luciferase and CFU (**Fig 4A and 4B**). In contrast, *Casp7/1/11*^*–/–*^macrophages are highly permissive for bacterial replication and phenocopy cells deficient for *Nlrc4*^*–/–*^and *Casp8/1/11/Ripk3*^*–/–*^(**Fig 4A and 4B**). These data support a role of CASP8 and CASP7 for restriction of bacterial replication in the absence of CASP1/11. As expected, *flaA* mutants evaded NAIP5/NLRC4 and effectively replicated in all macrophages regardless of the genotype (**Fig 4C and 4D**). We performed additional experiments comparing the replication of wild type *L*. *pneumophila* and isogenic *flaA* mutants in each macrophage genotype and found that *flaA* replicated significantly better than flagellin-positive bacteria in C57BL/6, *Casp1/11*^*–/–*^, *Casp7*^*–/–*^macrophages (**S3A–S3C Fig**). The *flaA* mutants phenocopy the wild type bacteria in *Nlrc4*^*–/–*^and *Casp8/1/11/Ripk3*^*–/–*^(**S3D and S3E Fig**) and *flaA* replicated slightly better than wild type bacteria in *Casp7/1/11*^*–/–*^macrophages (**S3F Fig**). These data indicate that NLRC4, CASP1, CASP8 and CASP7 are important for flagellin-mediated restriction of bacterial replication.

**Fig 5:**
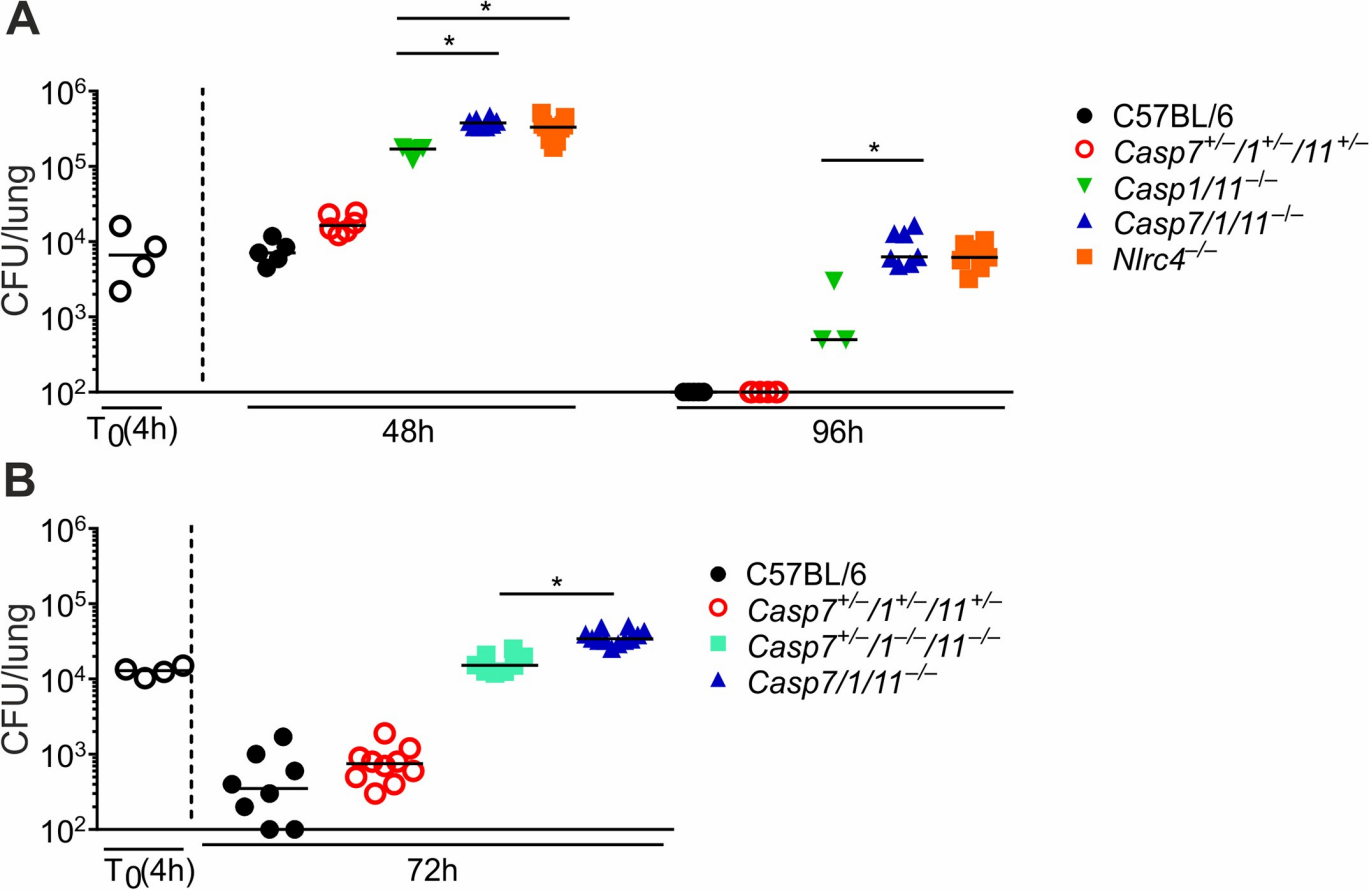
CASP7 is important for restriction of *L*. *pneumophila* replication *in vivo* in the absence of CASP1/11. (**A and B**) C57BL/6, *Casp1/11*^*–/–*^, *Casp7/1/11*^*–/–*^, *Casp7*^*+/–*^*/1*^*–/–*^*/11*^*–/–*^, *Casp7*^*+/–*^*/1*^*+/–*^*/11*^*+/–*^and *Nlrc4*^*–/–*^mice were infected intranasally with 10^5^ wild type *L*. *pneumophila*, lungs were harvested at the indicated time points and homogenates were plated for CFU determination. Each dot represents a single animal, and the horizontal lines represent the averages. *, *P*<0.05. Data are presented for one representative experiment of four (**A**) and two (**B**) experiments performed with similar results.

We have previously shown that *Nlrc4*^*–/–*^mice are more susceptible than *Casp1/11*^*–/–*^mice *in vivo* [22,36]. Thus, we tested if CASP7 accounts for the CASP1/11-independent mechanisms of restriction of bacterial replication. To test this, we infected mice with *L*. *pneumophila* and measured CFU in the lungs after 48 and 96hs. Strikingly, we found that *Casp7/1/11*^*–/–*^mice are as susceptible as the *Nlrc4*^*–/–*^mice and significantly more susceptible than *Casp1/11*^*–/–*^mice (**Fig 5A**). As expected the C57BL/6 and heterozygote control *Casp7*^*+/–*^*/1*^*+/–*^*/11*^*+/–*^are highly restrictive (**Fig 5A**). Next, we crossed the *Casp7/1/11*^*–/–*^x *Casp1/11*^*–/–*^to generate *Casp7*^*+/–*^*/1*^*–/–*^*/11*^*–/–*^progeny and infected these mice together with *Casp7/1/11*^*–/–*^mice. We confirmed that *Casp7/1/11*^*–/–*^mice are more susceptible than the *Casp7*^*+/–*^*/1*^*–/–*^*/11*^*–/–*^mice, suggesting that CASP7 accounts for restriction of *L*. *pneumophila* replication in the absence of CASP1/11 (**Fig 5B**).

### CASP7 and GSDMD operate downstream of CASP1 for restriction of *L*. *pneumophila* replication in macrophages and *in vivo*

**Fig 6:**
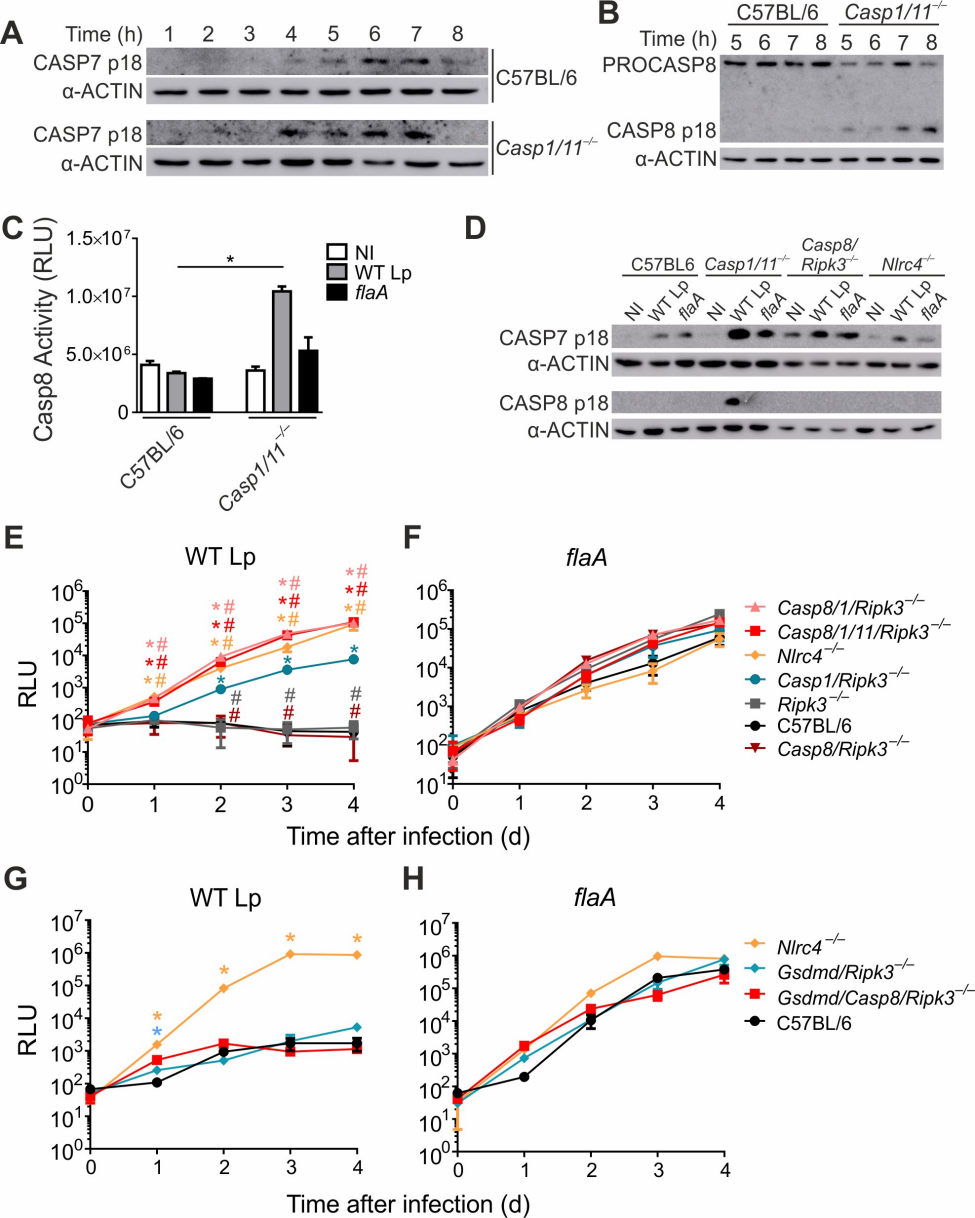
CASP7 is activated and contributes to restriction of *L*. *pneumophila* replication in the absence of CASP8. **(A–D)** Bone marrow-derived macrophages were left uninfected (NI) or were infected with wild type (WT Lp) or *flaA* mutants (*flaA*) *L*. *pneumophila* for the indicated time points to assay CASP7 and CASP8 activation. (**A**) Lysates from C57BL/6 and *Casp1/11*^*–/–*^macrophages infected with WT Lp for 1 to 8 h were assessed by Western blot using anti-Casp7 p18 antibodies and anti-α-actin. (**B**) C57BL/6 and *Casp1/11*^*–/–*^macrophages were infected with WT Lp for 5, 6, 7 and 8 h and Caspase-8 cleavage was measured by western blot anti-Casp8 p18 antibody and anti-α-actin. (**C**) C57BL/6 and *Casp1/11*^*–/–*^macrophages were infected for 8 h and CASP8 activation was measured using the Caspase-Glo 8 Assay kit. (**D**) Lysates from C57BL/6, *Casp1/11*^*–/–*^, *Casp8/Ripk3*^*–/–*^and *Nlrc4*^*–/–*^macrophages were assessed for CASP7 cleavage using anti-Casp7 p18 antibodies and anti-α-actin after 6 h infection. (**E–H**) Macrophages from C57BL/6, *Ripk3*^*–/–*^, *Casp1/Ripk3*^*–/–*^, *Casp8/Ripk3*^*–/–*^, *Casp8/1/Ripk3*^*–/–*^, *Casp8/1/11/Ripk3*^*–/–*^and *Nlrc4*^*–/–*^mice (**E-F**) or from C57BL/6, *Gsdmd/Ripk3*^*–/–*^, *Gsdmd/Casp8/Ripk3*^*–/–*^and *Nlrc4*^*–/–*^mice (**G-H**) were infected with wild type *L*. *pneumophila* (WT Lp) or *flaA* mutants (*flaA*) expressing luciferase at an MOI of 0.015 and bacterial replication was estimated by measurement of luminescence (RLU). Student’s *t* test. *, *P*<0.05 compared to C57BL/6. ^#^, *P*<0.05 compared to *Casp1/Ripk3*^*–/–*^. Data are presented for one representative experiment of three (**C**) and two (**A, B, E-H**) experiments performed with similar results.

Our data show that CASP7 operates downstream of CASP8 for restriction of *L*. *pneumophila* replication in *Casp1/11*^*–/–*^macrophages. Thus, we investigated if CASP7 is also activated in wild type macrophages. To test this we measured CASP7 cleavage in response to infection of C57BL/6 and *Casp1/11*^*–/–*^macrophages. We detected CASP7 processing both in C57BL/6 and *Casp1/11*^*–/–*^macrophages infected with wild type *L*. *pneumophila* (**Fig 6A**). In contrast, we only detected processing and activation of CASP8 in the *Casp1/11*^*–/–*^macrophages (**Fig 6B and 6C**). These data suggest that CASP7 can be activated in conditions where CASP8 is inactive, possibly by CASP1 as previously reported [17,28]. To test this hypothesis we performed experiments in macrophages that are deficient in CASP8 and sufficient in CASP1 and confirmed that CASP7 is cleaved in *Casp8/Ripk3*^*–/–*^cells (**Fig 6D**). In this experiment we detected a weak CASP7 cleavage in response to *flaA* and in *Nlrc4*^*–/–*^cells, suggesting the participation of another pathway for CASP7 activation.

We next performed growth curves in macrophages that are CASP1 positive and CASP8 negative to test if CASP1 activation is sufficient to trigger restriction of *L*. *pneumophila* replication. We found that *Casp8/Ripk3*^*–/–*^macrophages are as restrictive as C57BL/6 (and *Ripk3*^*–/–*^) macrophages (**Fig 6E**). As expected, *Casp1/Ripk3*^*–/–*^macrophages exhibit intermediate susceptibility and *Casp8/1/Ripk3*^*–/–*^, *Casp8/1/11/Ripk3*^*–/–*^macrophages are as susceptible as *Nlrc4*^*–/–*^macrophages (**Fig 6E**). The *flaA* mutants replicated in all macrophages tested, as expected (**Fig 6F**). These data confirm that CASP1 activation is sufficient to restrict *L*. *pneumophila* replication via the NAIP5/NLRC4 inflammasome despite the deficiency in CASP8.

In addition to CASP7, GSDMD is also downstream of CASP1. We tested by western blot if GSDMD is activated in response to *L*. *pneumophila* and found that GSDMD is cleaved in response to wild type *L*. *pneumophila* but not *flaA* mutants after 6 hs of infection (**S4 Fig**). *Gsdmd*^*–/–*^macrophages were included to control antibody specificity. GSDMD activation was found in C57BL/6 and *Asc*^*–/–*^macrophages but not in *Casp1/11*^*–/–*^and *Nlrc4*^*–/–*^(**S4 Fig**), indicating that GSDMD is cleaved by CASP1 independent of CASP8 via the NAIP5/NLRC4 inflammasome. We further generated mice double deficient in GSDMD and CASP8 to investigate if the CASP8-independent, CASP1-mediated restriction of *L*. *pneumophila* replication requires GSDMD. We found that *Gsdmd/Casp8/Ripk3*^*–/–*^cells are as restrictive as the *Gsdmd/Ripk3*^*–/–*^and C57BL/6 macrophages (**Fig 6G and 6H**). These data indicate that CASP1 activation triggers restriction of *L*. *pneumophila* replication in the absence of CASP8 and GSDMD, consistent with the participation of CASP7 downstream of CASP1.

**Fig 7:**
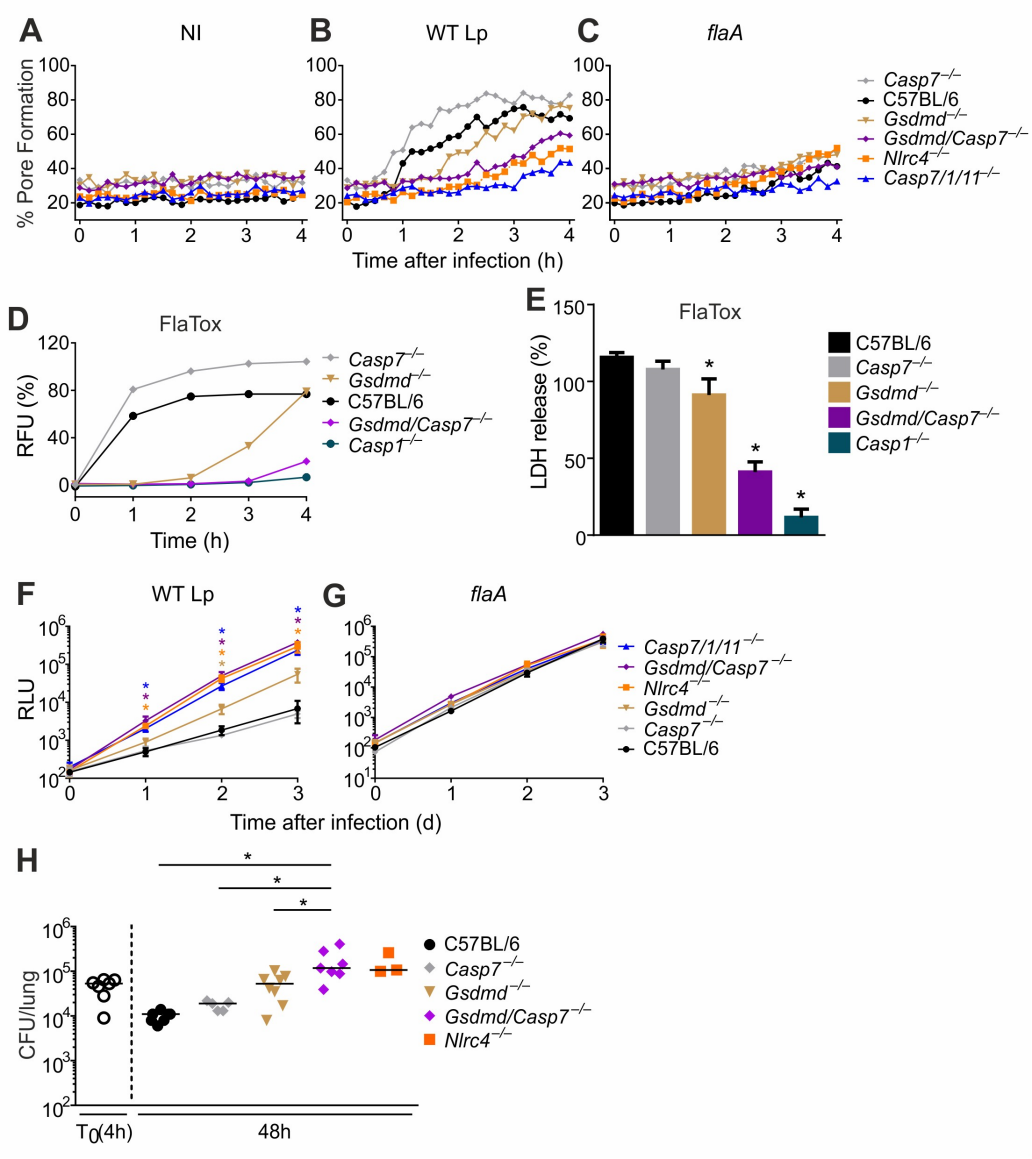
CASP7 and GSDMD are important for NAIP5/NLRC4/CASP1-dependent pore formation and restriction of *L*. *pneumophila* replication *in vivo*. **(A-C)** Bone marrow-derived macrophages from C57BL/6, *Casp7*^*–/–*^, *Gsdmd*^*–/–*^, *Gsdmd/Casp7*^*–/–*^, *Casp7/1/11*^*–/–*^and *Nlrc4*^*–/–*^mice were left uninfected (NI) or infected with wild type *L*. *pneumophila* (WT Lp) or *flaA* mutants (*flaA*) at an MOI of 10. Pore formation was assessed by Propidium Iodide (PI) uptake. Data are expressed as a percentage of PI uptake induced by Triton-X100. (**D** and **E**) Bone marrow-derived macrophages from C57BL/6, *Casp1*^*–/–*^, *Casp7*^*–/–*^, *Gsdmd*^*–/–*^and *Gsdmd/Casp7*^*–/–*^mice were stimulated with 4μg/ml Pa and 2μg/ml LFn-Fla (FlaTox). (**D**) The percentage of propidium iodide uptake was estimated by assessing the fluorescence (RFU). Data is shown as percentage of the total PI uptake (estimated using Triton-X100). (**E**) LDH release was assessed 4 hours after FlaTox treatment using CytoTox 96 Non-Radioactive Cytotoxicity Assay. (**F and G**) Bone marrow-derived macrophages from C57BL/6, *Casp7*^*–/–*^, *Gsdmd*^*–/–*^, *Gsdmd/Casp7*^*–/–*^, *Casp7/1/11*^*–/–*^and *Nlrc4*^*–/–*^mice were infected with WT Lp (**F**) or *flaA* (**G**) expressing luciferase at an MOI of 0.015 and bacterial replication was estimated by measuring the luminescence (RLU) of each well. *, *P*<0.05: compared to C57BL/6. Data are presented for one representative experiment of two (**A-C**), one (**D-E**) and two (**F-G**) experiments performed with similar results. (**H**) C57BL/6, *Casp7*^*–/–*^, *Gsdmd*^*–/–*^, *Gsdmd/Casp7*^*–/–*^and *Nlrc4*^*–/–*^mice were infected intranasally with 10^5^ wild type *L*. *pneumophila*, lungs were harvested at the indicated time points and homogenates were plated for CFU determination. Each dot represents a single animal, and the horizontal lines represent the averages. *, *P*<0.05. ns, *P*>0.05. Data presented in (**H**) are a pool of two independent experiments performed.

**Fig 8:**
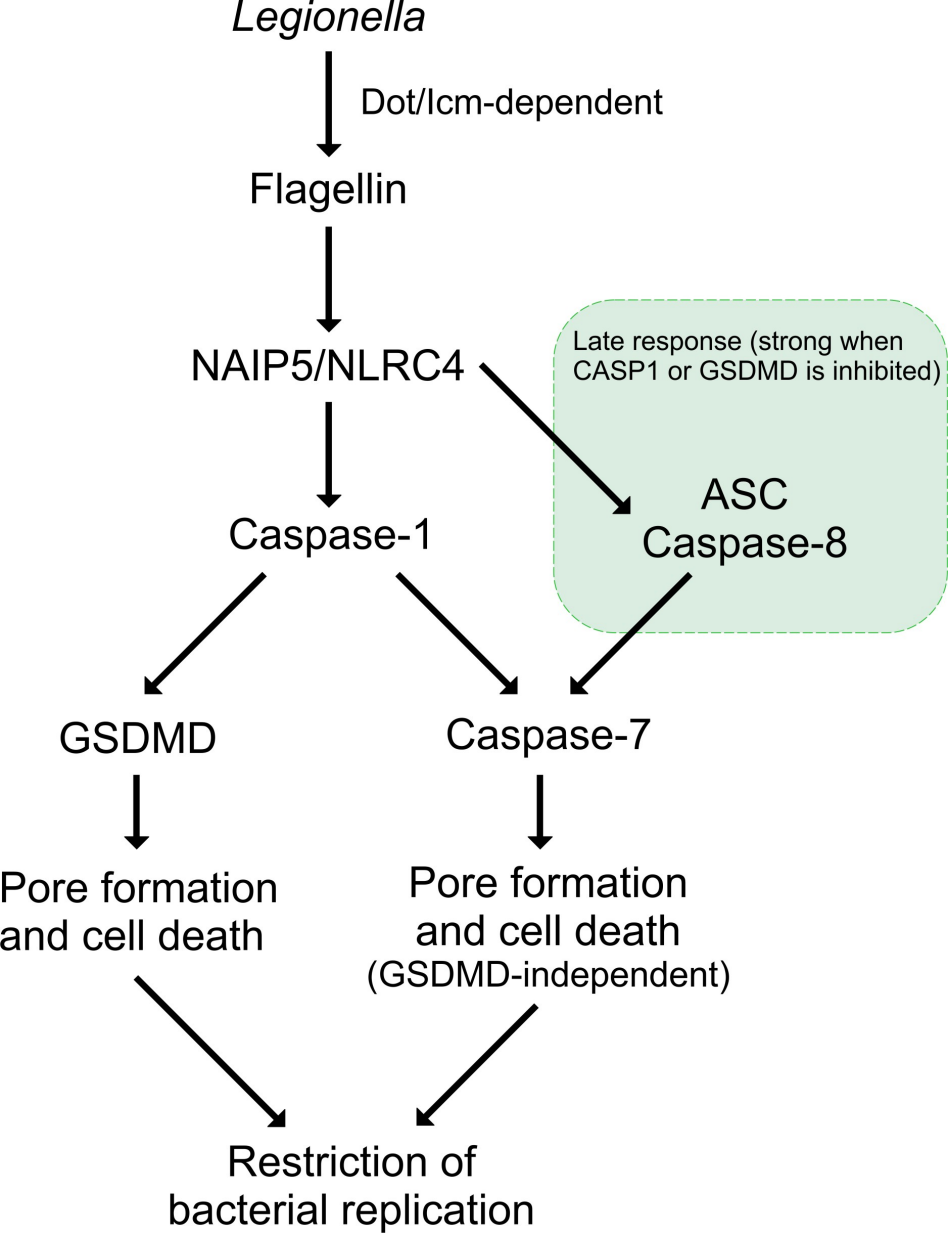
Schematic model illustrating that Gasdermin-D (GSDMD) and Caspase-7 are the key substrates of Caspase-1 and Caspase-8 downstream of the NAIP5/NLRC4 inflammasome.

Therefore, we reasoned that in wild type macrophages, CASP1 activates both GSDMD and CASP7 when the NAIP5/NLRC4 inflammasome is activated and that GSDMD and CASP7 can both mediate restriction of *L*. *pneumophila* independently of each other. The involvement of both CASP7 and GSDMD would explain why *Casp7*^*–/–*^or *Gsdmd*^*–/–*^singly deficient cells are able to restrict *L*. *pneumophila* replication in macrophages (**Figs 4A, 4B and 6G**). To test this hypothesis, we generated *Casp7/Gsdmd*^*–/–*^double deficient mice and tested the requirement of these molecules downstream of NAIP5/NLRC4 inflammasome. Initially, we tested pore formation in response to wild type *L*. *pneumophila* and *flaA* mutants and found that *Casp7/Gsdmd*^*–/–*^, *Casp7/1/11*^*–/–*^and *Nlrc4*^*–/–*^macrophages induced low pore formation in comparison to *Casp7*^*–/–*^, *Gsdmd*^*–/–*^and C57BL/6 macrophages (**Fig 7A–7C**). Of note, the pore formation assay performed with live *L*. *pneumophila* in presence of CASP11 may be difficult to interpret because LPS effectively triggers CASP11-mediated pore formation [25,27]. Therefore, we tested pore formation and LDH release in response to flagellin using FlaTox, a reagent that selectively activates NAIP5/NLRC4 without the confounding activation of CASP11 [37]. We found that cytosolic flagellin triggers a response that is CASP1-dependent and requires both CASP7 and GSDMD. This can be observed by pore formation (**Fig 7D)** and LDH release **(Fig 7E).** To evaluate the effect of CASP7 and GSDMD for restriction of *L*. *pneumophila* replication we performed macrophage infections and found that *Casp7/Gsdmd*^*–/–*^cells are significantly more permissive than the single KOs *Casp7*^*–/–*^and *Gsdmd*^*–/–*^(**Fig 7F and 7G**). *Casp7/Gsdmd*^*–/–*^macrophages phenocopied *Casp7/1/11*^*–/–*^and *Nlrc4*^*–/–*^for restriction of *L*. *pneumophila* replication (**Fig 7F**). We tested bacterial infection *in vivo* and found that the *Casp7/Gsdmd*^*–/–*^mice are as susceptible as the *Nlrc4*^*–/–*^mice (**Fig 7H**). In contrast, the *Casp7*^*–/–*^and *Gsdmd*^*–/–*^mice were significantly more restrictive, while still slightly more susceptible than C57BL/6, suggesting that these molecules are important for host resistance *in vivo*. Together, these data support a model that both CASP7 and GSDMD are required for restriction of *L*. *pneumophila* replication downstream of CASP1. When CASP1 or GSDMD is missing, CASP8 is activated in the NLRC4 inflammasome and triggers activation of CASP7, which also accounts for restriction of *L*. *pneumophila* replication in macrophages and in vivo (**Fig 8**).

## Discussion

CASP1 activation downstream of the NAIP5/NLRC4 inflammasome is critical for restriction of *L*. *pneumophila* replication in macrophages and in the lungs of infected mice [25,27], but the CASP1 substrates required for restriction of bacterial replication are still obscure. Previous work showed CASP7 is activated downstream of CASP1 [17,28] and is required for restriction of *L*. *pneumophila* replication via the NAIP5/NLRC4 inflammasome [28]. Although these data provide a direct link between CASP1 and CASP7, our experiments performed with macrophages and mice deficient only in CASP7 do not suggest an essential role of CASP7 for restriction of *L*. *pneumophila* replication *in vivo*. Indeed, we detected only a modest (less than one log) increase in bacterial loads in *Casp7*^*–/–*^compared to C57BL/6 macrophages during *L*. *pneumophila* growth curves in macrophages (**Figs 4A, 4B, 7F** and **7G**). In this study we sought to identify the redundant pathways that operate downstream of CASP1 for host resistance to infection. Our data support the hypothesis that redundancy between CASP7 and GSDMD explains the minor phenotypes of singly deficient *Gsdmd*^*–/–*^and *Casp7*^*–/–*^cells for restriction of *L*. *pneumophila* infection. This hypothesis is strongly supported by our data indicating that *Gsdmd/Casp7*^*–/–*^double-deficient mice are highly susceptible to *L*. *pneumophila* and phenocopy the *Nlrc4*^*–/–*^mice (**Fig 7**).

We and others have previously shown that CASP8 is also activated by the NAIP5/NLRC4 inflammasome, particularly when CASP1 or GSDMD is inhibited or missing [4,5,31]. As with CASP1, the CASP8 substrates required for bacterial restriction have been unclear. Interestingly, our data support a model indicating that CASP7 also operates downstream of CASP8 when the NAIP5/NLRC4 inflammasome is activated by cytosolic flagellin. We speculate that the kinetics of CASP8 activation in this inflammasome is slow compared with the quick and robust induction of pyroptosis that occurs via CASP1 and GSDMD. Therefore, CASP8 activation is preferentially detected in the absence of CASP1 and GSDMD. In these conditions, CASP7 is also activated downstream of CASP8 and our data using the *Casp7/1/11*^*–/–*^(and the *Casp8/1/11*^*–/–*^) mice and their macrophages strongly support the role of CASP7 downstream of CASP8 in the NAIP5/NLRC4 inflammasome. Together, our data indicate that CASP1 and CASP8 are the primary caspases activated by the NAIP5/NLRC4 inflammasome. Downstream of CASP1/8 we have now identified CASP7 and GSDMD as the key substrates required for restriction of bacterial replication (**Fig 8**). Our data therefore suggest that there is considerable redundancy built into the signaling outputs of the NAIP5/NLRC4 inflammasome: NAIP5/NLRC4 activates both CASP1 and CASP8; CASP1 can activate both GSDMD and CASP7; and CASP8 can activate CASP7 [38]. We speculate that this redundancy may be a mechanism for hosts to ensure responses even to pathogens that inhibit specific arms of the response. The identification of the critical substrates involved in the restriction of bacterial infection via the NAIP/NLRC4 inflammasomes provides important information for our understanding of the biology of these important platforms that operate for host protection against pathogenic bacteria. In response to *Yersinia spp*. infection, CASP8 was reported to trigger GSDMD activation [38,39]. Interestingly, it was recently reported that the GSDMD-mediated proinflammatory function of CASP8 is counteracted by CASP3-dependent cleavage and inactivation of GSDMD [40], suggesting that CASP3 suppresses GSDMD-mediated cell lysis during CASP8-induced apoptosis. We speculate that this may explain why we did not detect a CASP8-mediated GSDMD cleavage in *Casp1/11*^*–/–*^macrophages (**S4 Fig)** and a robust pore formation and LDH release in *Casp7/1/11*^*–/–*^(**Figs 3 and 7B**).

A question that arises from our studies is how CASP7 and GSDMD operate to restrict *L*. *pneumophila* replication. GSDMD is known to trigger pore formation and pyroptosis [20,21], a process that results in host cell death and thereby likely eliminates the intracellular replicative niche. In addition, pyroptosis has been proposed to result in formation of pore-induced intracellular traps (PITs) in macrophages infected with intracellular bacteria. The formation of PITs may help sequester bacteria and lead to their clearance by efferocytosis [41]. Our data demonstrate that CASP7 also promotes pore formation and host cell death. Although CASP7 appears to be involved in induction of cell death, the CASP7 substrates operating to trigger pore formation are still unclear. However, our data showing that CASP7 deficiency exerts a significant effect on bacterial replication even on a *Gsdmd*^–/–^background implies that CASP7 substrates other than GSDMD contribute to host defense [28,42]. It will be of interest to identify these substrates and their mechanisms of action in future studies.

## Material and methods

### Animals

Mice used in this study were breed and maintained in institutional animal facilities at FMRP/USP or at UC Berkeley. Mice used were C57BL/6 (Jax 000664), *Casp7*^*–/–*^(Jax 006237), *Nlrc4*^*–/–*^[43], *Casp1/11*^*–/–*^[44], *Asc*^*–/–*^[45], *Casp11*^*–/–*^[46], *Asc/Casp1/11*^*–/–*^[4], *Casp8*^*–/–*^[5], *Gsdmd*^*–/–*^[5], *Casp1*^*–/–*^[5], *Ripk3*^*–/–*^mice were originally from Xiaodong Wang [47] and backcrossed to C57BL/6 by Astar Winoto. Mice deficient in more than one gene not described above were generated in this study by intercrossing a F1 progeny of the parental strains.

### Ethics statement

The care of the mice was in compliance with the institutional guidelines on ethics in animal experiments; approved by CETEA (Comissão de Ética em Experimentação Animal da Faculdade de Medicina de Ribeirão Preto, approved protocol number 218/2014). CETEA follow the Brazilian national guidelines recommended by CONCEA (Conselho Nacional de Controle em Experimentação Animal). Animal experiments at UC Berkeley were approved by the institutional animal care and use committee. Mice were euthanized by CO_2_ asphyxiation with cervical dislocation as a secondary method.

### Bone marrow-derived macrophages

Bone marrow-derived macrophages were obtained as previously described [48]. Briefly, mice were euthanized and bone marrow cells were obtained from femurs and tibias. The cells were cultivated in RPMI 1640 (Gibco, Thermo Fisher Scientific, Massachussetts, USA) supplemented with 10–20% Fetal Bovine Serum (FBS) (Gibco) and 30% L929-Cell Conditioned Medium (LCCM) and 2 mM L-glutamine (Sigma-Aldrich) for 7 days, at 37°C, 5% CO_2_. In some experiments, instead of 20% LCCM it was used 10% of a conditional medium from 3T3 cells stably expressing mouse MCSF as a source of macrophage colony stimulation factor. Cells were detached with cold PBS, resuspended in RPMI 1640 supplemented with 10% FBS (R10) and plated as indicated. For all in vitro experiments, the plates were centrifuged at 300 x g for 5 min, room temperature, after cell plating and infection, to ensure homogeneous adherence of cells and infection synchronization, respectively. Incubation of non-infected and infected cells was done at 37°C, 5% CO_2_.

### Bacteria culture and preparation

*Legionella pneumophila* strains used were JR32 and isogenic mutants for *flaA* and *fliI* as previously described [12,22]. For some experiments, *L*. *pneumophila* strains stably expressing the *Photorhabdus luminescens luxCDABE* operon were used. Bacteria were cultured in Charcoal-Yeast Extract Agar (CYE, 10 g/L 4-morpholinepropanesulfonic acid [MOPS], 10 g/L Yeast extract, 15 g/L technical agar, 2 g/L activated charcoal, supplemented with 0.4 g/L L-cysteine and 0.135 g/L Fe(NO_3_)_3_) at 35–37°C, for 4 days from frozen stocks. Single colonies were streaked on fresh plates and allowed to grow for another 2 days. For in vitro infections, bacteria grown on solid plates were resuspended in autoclaved distilled water and diluted on RPMI as indicated. For in vivo infections, bacteria grown on solid plates were resuspended in autoclaved distilled water and diluted in Phosphate-buffered saline (PBS) as needed.

### Bacterial replication in macrophages

Experiments to quantify bacterial CFU in macrophages were made in 24-well plates. A total of 2 x 10^5^ macrophages were plated per well in R10 and incubated overnight. The medium was replaced with the bacterial suspension in R10 with the indicated multiplicities of infection (MOIs) for 1hr before being replaced again by fresh R10 media. At the indicated time points, the supernatants were collected, cells were lysed with autoclaved distilled water and the lysate was added to the supernatants. Dilutions were plated on CYE and incubated for 4 days for counting of colony-forming units (CFU). Experiments to measure bacterial replication using a luminescence-based replication assays were made as previously described [33]. Briefly, 10^5^ macrophages/well were plated on white 96-well plates and incubated overnight. The medium was replaced with the bacterial suspension in R10 with an MOI of 0.01 or 10. At the indicated time points, luminescence emission was measured at 470 nm with a Spectra-L plate reader (Molecular Devices, California, USA).

### Cell toxicity assay by LDH release

5 x 10^5^ macrophages/well were plated on 24-well plates in R10 and incubated overnight. The medium was replaced with the bacterial suspension (estimated to reach an MOI of 10) in RPMI without Phenol Red (3.5 g/L HEPES, 2 g/L NaHCO_3_, 10.4 g/L RPMI without Phenol Red, 1% glutamine, pH 7.2) and incubated for 7h. The supernatant was collected and LDH release was measured using CytoTox 96® Non-Radioactive Cytotoxicity Assay (Promega, Winsconsin, USA) following the manufacturer’s instructions.

### Pore formation assay

For estimation of pore formation, 10^5^ macrophages/well were plated on black, clear bottom 96-well plates in R10 and incubated overnight. The medium was replaced with the bacterial suspension (estimated to reach an MOI of 10) in RPMI without Phenol Red and low on NaHCO_3_ (2 g/L HEPES, 0.38 g/L NaHCO_3_ 10.4 g/L RPMI without Phenol Red, 1% glutamine, 2% FBS, pH 7.2), 1:1000 rabbit anti-*Legionella* antibody and 6 μL/mL propidium iodide (PI). PI was excited at 538 nm and emission was measured at 617 nm with a SpectraMax plate reader (Molecular Devices, California, USA). Triton-X100 1.3% was used as a positive control and for normalization.

### Caspase-8 activation assay

A total of 10^5^ cells/well were plated on white 96-well plates in R10 and incubated overnight. The medium was replaced with the bacterial suspension in RPMI without Phenol Red and low on NaHCO_3_ (2 g/L HEPES, 0.38 g/L NaHCO_3_ 10.4 g/L RPMI without Phenol Red, 1% glutamine, 2% FBS, pH 7.2) with an MOI of 10 and the plate was incubated for 8h. The supernatants were collected and Caspase-8 activation was measured using Caspase-Glo 8 Assay (Promega, Winsconsin, USA) following the manufacturer’s instructions.

### Western blotting

To measure caspase and GSDMD cleavage by western blot, 10^6^ cells/well were plated on 24-well plates (for CASP7 and CASP3) or 48-well plates (for CASP8 and GSDMD) in R10 and incubated overnight. The medium was replaced with the bacterial suspension in R10 with an MOI of 10 and the plate was incubated for the indicated times. The supernatants were discarded (for CASP7 and CASP3) or collected (for CASP8 and GSDMD) and cells were lysed with 50 μL of RIPA supplemented with protease inhibitor (Complete Protease Inhibitor Cocktail, Roche, Basel, Switzerland). For CASP8 and GSDMD, lysates were added to the supernatants. Samples were immediately sonicated for 10 min and frozen at -80°C until analysed. A total of 50 μg of protein from each sample were run on a 15% acrylamide gel, transferred onto a nitrocellulose membrane and the membranes were incubated overnight, at 4°C under mild agitation with Anti-cleaved CASP7 antibody (rabbit) (Cell Signaling Technologies, Massachussetts, USA) diluted 1:1000 in 5% BSA in TBS 1X with 0.01% Tween; or Caspase-8 (D35G2) Rabbit mAb (Cell Signaling Technologies, Massachussetts, USA) diluted 1:1000 in 5% non-fat dry milk in TBS 1X with 0.01% Tween; Caspase-3 antibody (#9662, Cell Signaling Technologies, Massachussetts, USA) diluted 1:1000 in 5% non-fat dry milk in TBS 1X with 0.01% Tween; or a rat monoclonal antibody against GSDMD (GN20-13, Genentech) diluted 1:1000 in 5% non-fat dry milk in TBS 1X with 0.01% Tween. Actin was stained with rabbit anti-α-actin (#A2066, Sigma-Aldrich, Missouri, USA) diluted 1:5000 in 5% non-fat dry milk in TBS 1X with 0.01% Tween. The membranes were incubated for 1h with goat anti-rabbit or anti-rat secondary antibodies (Sigma-Aldrich, Missouri, USA) and analyzed using ECL™ Prime Western Blotting System (GE Healthcare, Illinois, EUA) and an Amersham Imager 600 (GE Healthcare, Illinois, EUA). Bands were quantified using ImageJ.

### In vivo replication assays

All mice were matched by sex and age (all were at least 8 weeks old at the time of infection) and were in a C57BL/6 mouse genetic background. For the in vivo experiments, approximately 5–7 mice per group were used, as indicated in the figures. Mice were infected intranasally with 10^5^ bacteria contained in 40 μL of PBS. The animals were anesthetized with ketamine (50mg/kg) and xylazine (10mg/kg) intraperitoneally and infected. At the indicated time points, the lungs were harvested and macerated for 30 seconds in 5 mL of autoclaved distilled water using a tissue homogenizer (Power Gen 125; Thermo Scientific). Dilutions were plated on CYE + 10 μg/mL of streptomycin and plates were incubated for 4 days at 37°C for CFU counting.

### Statistical analysis

The data were plotted and analyzed using GraphPad Prism 5.0 software. The statistical significance was calculated using the Student’s t-test or analysis of variance (ANOVA). Differences were considered statistically significant when *P* was <0.05, as indicated by an asterisk in the figures.

## Supporting information

S1 Fig CASP8 is activated in the absence of CASP1/11 and it is important for restriction of *L*. *pneumophila* replication in macrophages. Macrophages were infected with Lp02 WT *L*. *pneumophila* (**A**), Lp02 *flaA* mutants (**B**) or Lp02 *dotA*^*−*^mutants (**C**) expressing luciferase at an MOI of 0.015 and bacterial replication was estimated by measuring the luminescence (RLU) of each well over 4 days of infection. Statistical significance was calculated using Student’s *t* test. *, *P*<0.05: compared to C57BL/6. ^#^, *P*<0.05: compared to *Casp1/11/Ripk3*^*–/–*^.
(TIF)

S2 Fig CASP7 is activated downstream of CASP8 in the NAIP5/NLRC4 inflammasome in immortalized macrophages. Immortalized macrophages from *Casp1/11*^*–/–*^, *Asc/Casp1/11*^*–/–*^, *Casp8/1/11/Ripk3*^*–/–*^and *Casp7/1/11*^*–/–*^mice were left uninfected or infected with wild type *L*. *pneumophila* (WT Lp, grey bars) or *flaA* mutants (black bars) at an MOI of 10 for 8 hours. Caspase-8 activation was measured by western blot using anti-Casp8 p18 antibody. Bands were quantified using ImageJ.
(TIF)

S3 Fig CASP7 is important for restriction of *L*. *pneumophila* replication in vivo in the absence of CASP1/11. Bone marrow-derived macrophages from C57BL/6, *Casp7*^*–/–*^, *Casp1/11*^*–/–*^, *Casp7/1/11*^*–/–*^,*Casp8/1/11/Ripk3*^*–/–*^and *Nlrc4*^*–/–*^mice were infected with wild type *L*. *pneumophila* or *flaA* mutants. Macrophages were infected at an MOI of 0.015 and bacterial replication was assessed by measurement of luminescence (RLU) emitted by luciferase-expressing bacteria. Statistical significance was calculated using Student’s t test. *, *P*<0.05.
(TIF)

S4 Fig GSDMD cleavage in response to *L*. *pneumophila* infection for 6 h requires flagellin, NLRC4 and CASP1/11. Macrophages from C57BL/6, *Casp1/11*^*–/–*^, *Nlrc4*^*–/–*^, *Asc*^*–/–*^and *Gsdmd*^*–/–*^mice were left uninfected (NI) or infected with wild type *L*. *pneumophila* (WT Lp) or *flaA* mutants (*flaA*) at an MOI of 10 for 6 hs. GSDMD cleavage in the supernatants plus cell lysates were measured by western blot using the anti-GSDMD antibody.
(TIF)
