# Supplementary figures and images for: Gasdermin-D and Caspase-7 are the key Caspase-1/8 substrates downstream of the NAIP5/NLRC4 inflammasome required for restriction of Legionella pneumophila
Source: PLoS Pathog. 2019 Jun 28;15(6):e1007886. doi: 10.1371/journal.ppat.1007886 (PMC6622555; doi:10.1371/journal.ppat.1007886)

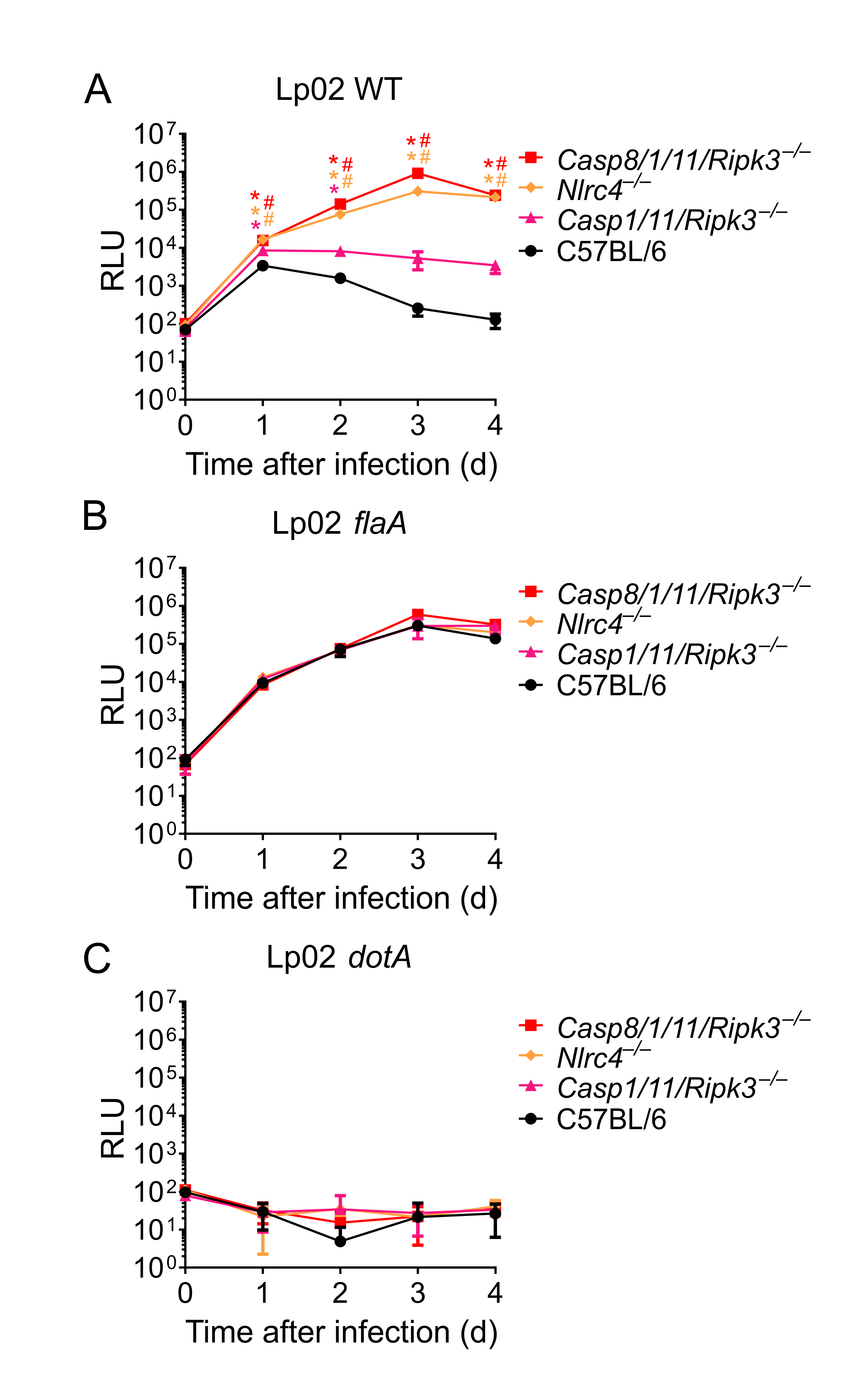

Supplement: S1 Fig — Macrophages were infected with Lp02 WT L. pneumophila (A), Lp02 flaA mutants (B) or Lp02 dotA−mutants (C) expressing luciferase at an MOI of 0.015 and bacterial replication was estimated by measuring the luminescence (RLU) of each well over 4 days of infection. Statistical significance was calculated using Student’s t test. *, P<0.05: compared to C57BL/6. #, P<0.05: compared to Casp1/11/Ripk3–/–. (TIF) [file ppat.1007886.s001.tif]

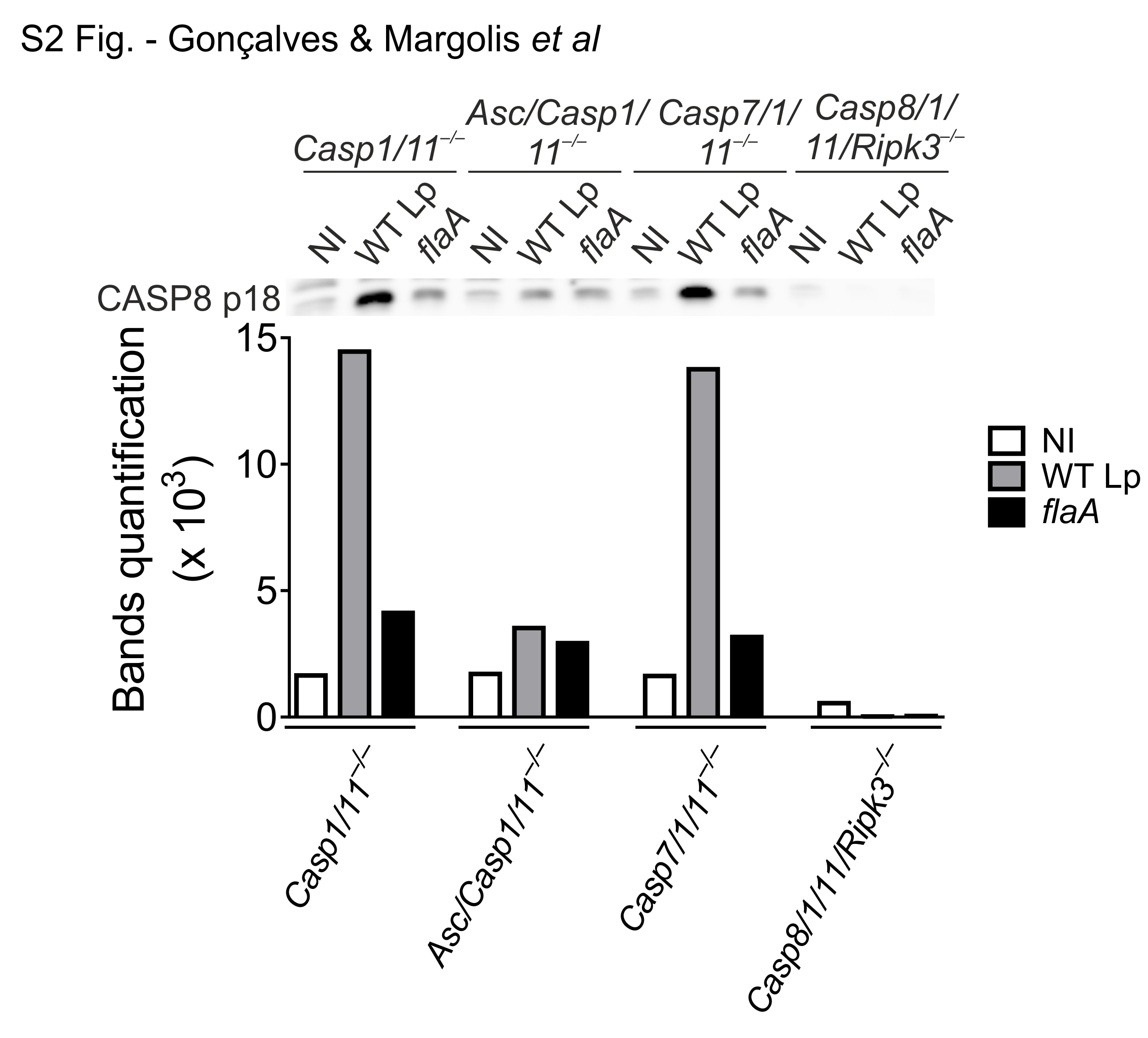

Supplement: S2 Fig — Immortalized macrophages from Casp1/11–/–, Asc/Casp1/11–/–, Casp8/1/11/Ripk3–/–and Casp7/1/11–/–mice were left uninfected or infected with wild type L. pneumophila (WT Lp, grey bars) or flaA mutants (black bars) at an MOI of 10 for 8 hours. Caspase-8 activation was measured by western blot using anti-Casp8 p18 antibody. Bands were quantified using ImageJ. (TIF) [file ppat.1007886.s002.tif]

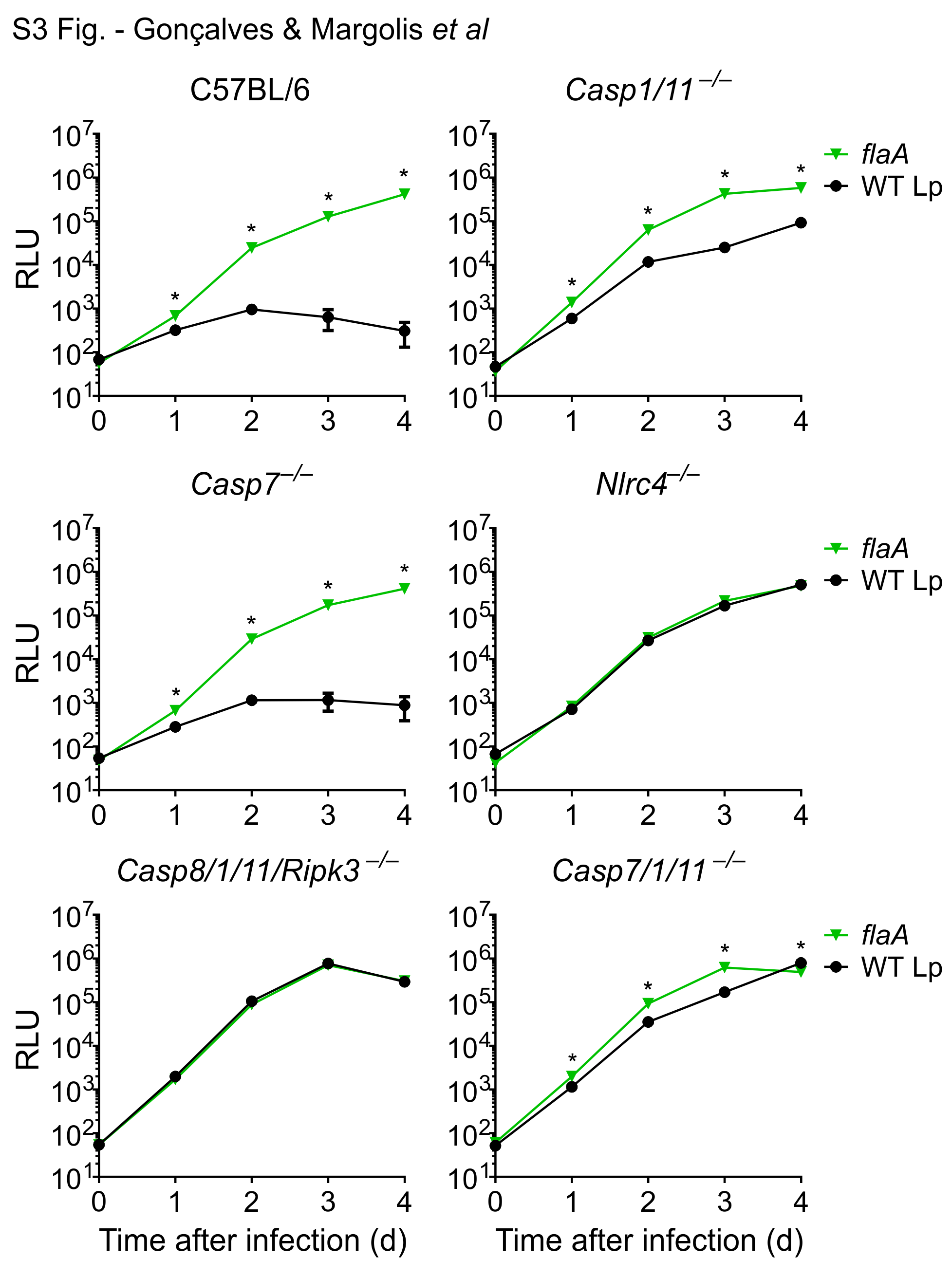

Supplement: S3 Fig — Bone marrow-derived macrophages from C57BL/6, Casp7–/–, Casp1/11–/–, Casp7/1/11–/–,Casp8/1/11/Ripk3–/–and Nlrc4–/–mice were infected with wild type L. pneumophila or flaA mutants. Macrophages were infected at an MOI of 0.015 and bacterial replication was assessed by measurement of luminescence (RLU) emitted by luciferase-expressing bacteria. Statistical significance was calculated using Student’s t test. *, P<0.05. (TIF) [file ppat.1007886.s003.tif]

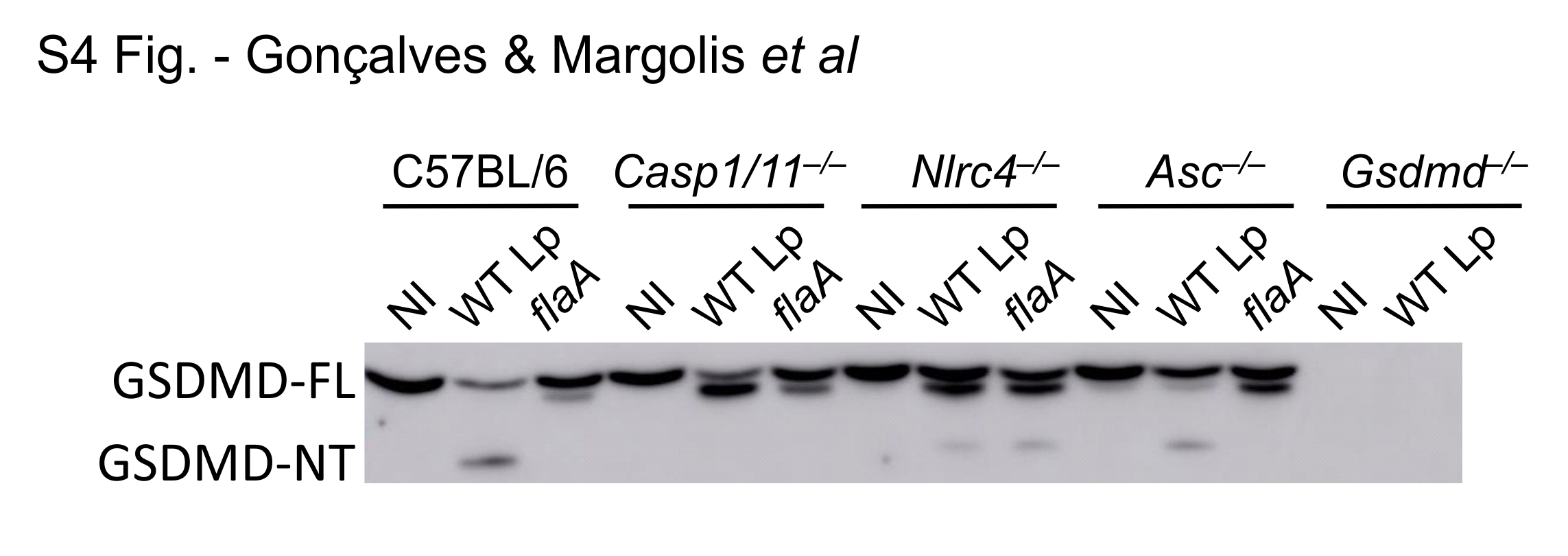

Supplement: S4 Fig — Macrophages from C57BL/6, Casp1/11–/–, Nlrc4–/–, Asc–/–and Gsdmd–/–mice were left uninfected (NI) or infected with wild type L. pneumophila (WT Lp) or flaA mutants (flaA) at an MOI of 10 for 6 hs. GSDMD cleavage in the supernatants plus cell lysates were measured by western blot using the anti-GSDMD antibody. (TIF) [file ppat.1007886.s004.tif]
